# Long‐term care and the coronavirus disease 2019 challenge in Japan

**DOI:** 10.1002/jgf2.366

**Published:** 2020-08-19

**Authors:** Shiho Amagasa, Hiroyuki Kojin, Shigeru Inoue

**Affiliations:** ^1^ Department of Preventive Medicine and Public Health Tokyo Medical University Tokyo Japan; ^2^ Department of Quality and Safety management Graduate Faculty of Interdisciplinary research Faculty of medicine University of Yamanashi Yamanashi Japan

To the Editor,

The spread of the coronavirus disease 2019 (COVID‐19) poses a potential threat to long‐term care (LTC) in Japan. It is estimated that up to half of those who died of COVID‐19 in Europe were nursing home residents,^1^ and older adults, particularly those with medical conditions, are vulnerable to COVID‐19. Therefore, many LTC services have temporarily suspended operations to reduce the risk of infection. Although it is important to maintain LTC service even in such a difficult situation, the widespread suspension of LTC services may be inevitable considering the tragic circumstances unfolding domestically^2^ and internationally,^1^ and it could lead to the collapse of LTC.

In Japan, there is a rising concern regarding a potential impact of suspension of day‐care services on older adult's health. At least 909 LTC services (858 are day‐care and 51 are home‐visit services) have temporarily suspended operations as of April 20, 2020, due to the risk of infection.^3^ Day‐care services include a wide variety of programs and services provided at LTC facilities (eg, rehabilitation, diet, exercise, and bathing). The use of day‐care services plays a significant role in the mental health, physical functioning, social connections, and quality of life of older adults.^4^ It also offers a respite for caregivers, including family.

Japan's Ministry of Health, Labour and Welfare has recommended that alternative LTC services (eg, home‐visit services) be provided where LTC facilities and day‐care services have been suspended. However, the lack of care workers is making it impossible to maintain LTC services. LTC workers are also at a higher risk of infection due to the shortage of personal protective equipment.^5^ In addition, LTC workers are stopping their jobs.^5^ This has led to a deterioration in the management of LTC services, and many LTC businesses have fallen into a negative spiral. According to a national survey by the Japan Federation of Kaigo Business Providers, 82% day‐care business has been financially impacted, and 14% are likely to be impacted.^5^ To minimize the disruption in LTC services during the COVID‐19 pandemic, strong financial support for LTC services by the government will be one of the key measures to rapidly restart this service industry.

Under the LTC system as a part of national insurance for universal health coverage, public LTC prevention plans have focused on promoting social participation and preventing the social isolation of older adults. However, due to severe restrictions on going out and social participation to reduce infection risk, the number of older adults with deteriorated physical functioning and mental health is likely to increase during the COVID‐19 pandemic, particularly in aging countries. A variety of resources (eg, web‐based approach) are provided around the world to help prevent the deterioration of older adults' health.^6^, ^7^ More research is needed to understand these concerns and to identify what policies and interventions are most effective, especially with the post‐COVID‐19 era in mind.

## CONFLICT OF INTEREST

The authors have stated explicitly that there are no conflicts of interest in connection with this article.
